# Continuous glucose monitoring as a tool in early-stage type 1 diabetes

**DOI:** 10.1007/s00125-026-06707-4

**Published:** 2026-03-09

**Authors:** Alice L. J. Carr, Hannah Sutton, Rikke M. Agesen, Ezio Bonifacio, Emanuele Bosi, Pieter Gillard, Rabbi Swaby, Jurgen Vercauteren, Rachel E. J. Besser

**Affiliations:** 1 Alberta Diabetes Institute, University of Alberta, Edmonton, AB Canada; 2 Diabetes and Inflammation Laboratory, Centre for Human Genetics, Nuffield Department of Medicine, Bukhman Centre for Research Excellence in Type 1 Diabetes, NIHR Oxford Biomedical Research Centre, University of Oxford, Oxford, UK; 3 Novo Nordisk A/S, Søborg, Denmark; 4 Center for Regenerative Therapies Dresden (CRTD), Faculty of Medicine, Technische Universität Dresden, Dresden, Germany; 5 Diabetes Research Institute, San Raffaele Hospital, Milan, Italy; 6 San Raffaele Vita Salute University, Milan, Italy; 7 Department of Endocrinology, University Hospitals Leuven, Leuven, Belgium; 8 Research Foundation Flanders-FWO, Leuven, Belgium; 9 Department of Chronic Diseases and Metabolism, KU Leuven, Leuven, Belgium; 10 Department of Paediatrics, University of Oxford, Oxford, UK

**Keywords:** Continuous glucose monitoring, Progression, Review, Stage 1 diabetes, Stage 2 diabetes, Type 1 diabetes

## Abstract

Continuous glucose monitoring (CGM) has transformed diabetes management by providing continuous, real-time insights into glucose dynamics, while enhancing the lived experience of individuals with type 1 diabetes. In established type 1 diabetes, CGM-derived measures of glucose management, such as time in range, time above range, time below range and glycaemic variability, have become integral tools to optimise therapy, reduce episodes of hypoglycaemia and guide clinical decision-making. More recently, CGM has emerged as a promising tool to detect early hyperglycaemia and other glucose abnormalities in individuals with early-stage type 1 diabetes, for whom current screening and staging methods, including fasting glucose, HbA_1c_ and the OGTT, remain limited by episodic sampling, participant burden and variable reproducibility. This review examines the rationale, evidence and practical considerations for integrating CGM into early-stage type 1 diabetes research and clinical frameworks. We discuss its potential to complement existing metabolic and immunological markers, as well as the technical, analytical and regulatory challenges that must be addressed for CGM to serve as a reliable tool for screening, staging and monitoring and as a clinical endpoint in early-stage type 1 diabetes.

**Graphical Abstract:**

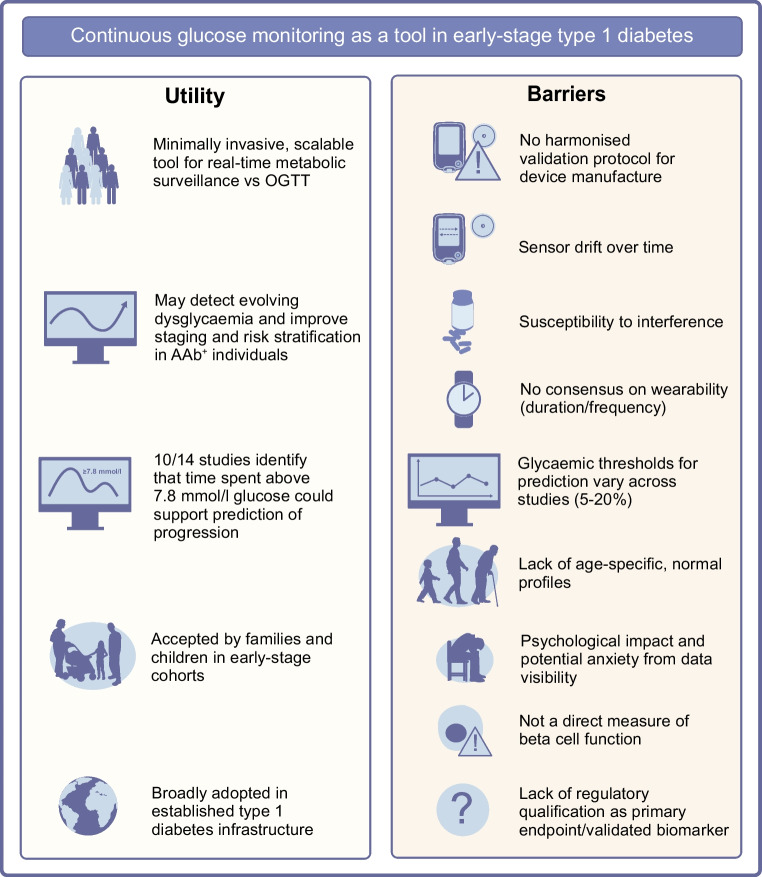

**Supplementary Information:**

The online version contains a slideset of the figures for download available at 10.1007/s00125-026-06707-4.

## Introduction

The rising global incidence of type 1 diabetes combined with the persistently high and, in some regions, increasing rates of diabetic ketoacidosis (DKA) underscore the urgent need for targeted public health strategies [1–3]. Screening and monitoring programmes to detect type 1 diabetes in the early stages of disease mitigate the risk of DKA [4].

Symptomatic type 1 diabetes (stage 3) occurs when an insufficient number of functional beta cells remain to maintain glucose homeostasis [5, 6]. This clinical presentation is preceded by progressive beta cell loss driven by autoimmune destruction over months or years [7–10]. This offers an opportunity for earlier detection and intervention, through timely monitoring before insulin is initiated. With the recent regulatory approval of teplizumab, the first disease-modifying drug for stage 2 type 1 diabetes, and other emerging interventions [11], emphasis is growing on identification of diabetes before stage 3. Currently, the oral glucose tolerance test (OGTT) remains the gold standard for staging and monitoring type 1 diabetes [7–9, 12, 13], but its invasiveness, cost, variability and poor tolerability [14–17] highlight the need for alternatives.

Interest is growing in leveraging the dynamic, real-time insights of continuous glucose monitoring (CGM) data to detect, stage and guide intervention in early-stage type 1 diabetes. Screening for type 1 diabetes is now increasingly offered not only to first-degree relatives but also to the general population, to identify individuals with early-stage disease, defined as the confirmed presence of two or more autoantibodies. In this context, CGM could help refine risk stratification, confirm early dysglycaemia or track disease progression, and support the timely identification of individuals who may benefit from disease-modifying therapies.

However, expanding the context of use of CGM in early-stage type 1 diabetes will require robust evidence. For example, CGM-derived glucose patterns and measures of variability may function as prognostic biomarkers by informing the likelihood or timing of disease progression. These metrics may also serve as predictive biomarkers, identifying individuals more likely to respond to an intervention when treatment response is the endpoint [18]. Recent reviews have highlighted the potential for CGM-derived diagnostic thresholds to assist in diagnosing early dysglycaemia and defining stage 2 disease [19, 20]. In this review, we synthesise current evidence on the use of CGM in early-stage type 1 diabetes, examining its potential applications in screening, staging and monitoring, and outline the key technical, analytical and regulatory considerations that will determine its future use and that must be addressed for its broader adoption.

## Established staging and monitoring tools in early-stage type 1 diabetes

### Islet autoantibodies

The presence of multiple islet autoantibodies remains a robust biomarker for early-stage type 1 diabetes, with the presence of two or more islet autoantibodies indicating that the autoimmune process has begun and the lifetime risk of progression to clinical (stage 3) type 1 diabetes approaches 100% [7–9, 21, 22]. The number, titre and type of autoantibody further inform the rate of progression [21, 23–25].

### Glycaemic markers: OGTT and HbA_1c_

In addition to the presence of two or more islet autoantibodies, staging of preclinical disease is currently guided by the level of glucose tolerance assessed by an OGTT and/or HbA_1c_ [7–9].

Early-stage type 1 diabetes is characterised by distinct metabolic phases in which individuals may alternate between dysglycaemia (stage 2) and normoglycaemia (stage 1), complicating staging and risk stratification. In TrialNet, stage 2 eligibility requires two abnormal serial assessments, defined by impaired fasting glucose (6.1–6.9 mmol/l), impaired glucose tolerance at 120 min (7.8–11.0 mmol/l) or elevated intermediate OGTT values (≥11.1 mmol/l) [26]. It is important to note that these criteria derive from the strict dysglycaemia definition used in the pivotal TrialNet TN-10 study [27], which formed the basis for teplizumab approval. The ADA applies broader fasting glucose (5.6–6.9 mmol/l) and HbA_1c_ (39–46 mmol/mol [5.7–6.4%]) thresholds and only the 120 min OGTT value, without intermediate values [9]. The Fr1Da study showed a higher 2 year progression risk when using TrialNet criteria (62.9%) than when using ADA criteria (32.2%) [28].

An increase in HbA_1c_ levels has also been suggested as a specific indicator of progression to stage 3 type 1 diabetes [29–31], with a ≥10% rise over 3–12 months suggestive of increased risk of progression to stage 3 within 2 years [32]. However, HbA_1c_ is relatively insensitive, lags behind real-time glucose values and is influenced by multiple factors [33]. Consensus guidance therefore recommends combining ADA and TrialNet definitions to robustly distinguish normo- and dysglycaemia for staging accuracy, alongside metabolic monitoring, education on diabetes/DKA symptoms, and psychosocial support [13].

## Rationale for evaluating CGM in early-stage type 1 diabetes

There is a growing momentum towards the use of CGM in early-stage type 1 diabetes to address key unmet needs required for effective general population screening programmes.

### The need for continuous surrogate markers of beta cell health

The decline in functional beta cell mass due to autoimmune destruction typically occurs months or years before clinical diagnosis [34, 35]. The evolution of disease onset is heterogeneous in rate and metabolic manifestation [36–38]; hence, beta cell function or ‘health’ is best conceptualised by the disposition index, which captures the hyperbolic relationship between insulin secretion and sensitivity [39–42].

The AUC of C-peptide during dynamic testing (i.e. OGTT, mixed-meal tolerance test [MMTT] or intravenous glucose tolerance test [IVGTT]) is widely accepted as a surrogate marker of beta cell function and as a trial endpoint [27, 43–47]. The earliest detectable abnormality, loss of the first-phase insulin response (FPIR), is most sensitively measured using a hyperglycaemic clamp or IVGTT [39, 48–50], although these methods are considered impractical for large-scale use.

Additional biomarkers such as the proinsulin:C-peptide ratio [51–53] and pragmatic composite indices such as the BETA-2 score [54–57], M120 risk score [58], progression likelihood score [36], Quantitative Risk Score [59], Index60 and Diabetes Prevention Trial–Type 1 Risk Score (DPTRS) [60–63] have shown value in tracking beta cell decline, predicting progression and identifying early treatment response [64]. More complex modelling approaches such as the oral minimal model [65, 66] allow estimation of insulin sensitivity and beta cell responsivity from OGTT data, possibly offering greater granularity in dissecting metabolic heterogeneity in the early stages of disease [38, 39, 67].

However, even the most sophisticated indices are constrained by their reliance on intermittent OGTT testing, making it difficult to pinpoint the timing of beta cell functional changes [68]. Cohort studies demonstrate that autoantibody-positive individuals can move between normoglycaemia (stage 1) and dysglycaemia (stage 2) [69, 70]. Longitudinal data from TrialNet showed that 36% of individuals with an abnormal OGTT reverted to normoglycaemia at their next assessment [71]. It is plausible that many of these periods of metabolic recovery and decline are masked by the limitations of conventional testing intervals and tools.

### The need for minimally invasive and scalable surveillance

To enable earlier detection of type 1 diabetes and intervention at scale, screening must be delivered to the general population, as the majority (>85%) of affected individuals do not have an affected first-degree relative [12, 72, 73]. However, the gold standard OGTT [7–9, 12] is invasive, time-consuming and often distressing for young children [14, 74], requiring preparatory carbohydrate loading, prior fasting, cannulation, ingestion of a glucose load and multiple blood draws over 2 h. In addition, practical limitations such as the need for immediate processing to avoid glycolysis and sample degradation, the need for specialised facilities, and poor reproducibility necessitate repeated monitoring in both children and adults [14–17, 74], limiting scalability. CGM offers a minimally invasive alternative for disease surveillance and risk stratification [13, 19, 75] in both adults and very young children [76]. Moreover, as it is widely recommended for monitoring in established type 1 diabetes, many diabetes professionals have experience in its use.

**Fig. 1:**
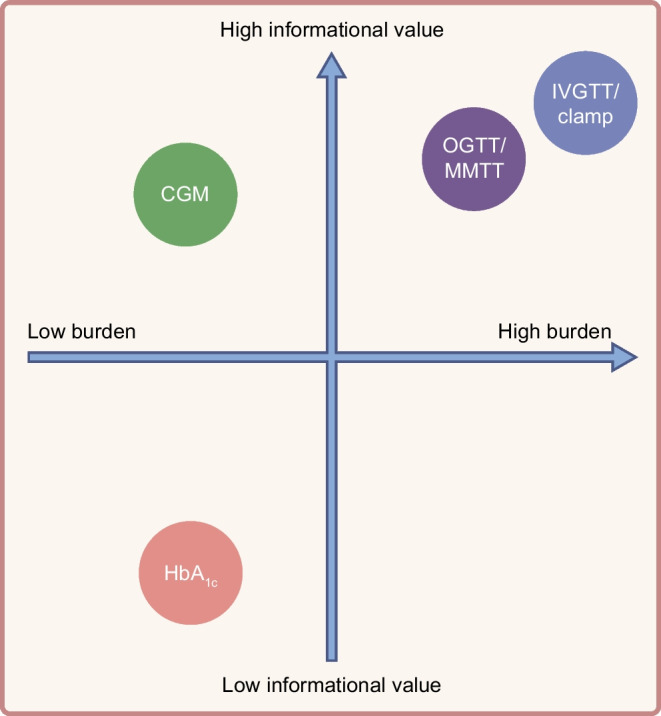
Schematic of the relative burden (invasiveness, cost, tolerability, logistics) and informational value (capturing disease staging, beta cell function and risk of progression) of current metabolic monitoring tools in early-stage type 1 diabetes. This figure is available as part of a downloadable slideset

As general population screening initiatives emerge worldwide [77–81], follow-up strategies must balance informative value with accessible, low-burden monitoring technologies (Fig. 1) to improve early detection, risk stratification and long-term adherence.

## Current recommendations and clinical implementation of CGM in type 1 diabetes

Until recently, monitoring of clinical type 1 diabetes relied primarily on self-monitoring of blood glucose (SMBG). In 2022, the UK National Institute for Health and Care Excellence marked a major shift by recommending CGM for all individuals with type 1 diabetes [82, 83]. Similar updates from the ADA, International Society for Paediatric and Adolescent Diabetes (ISPAD) and American Association of Clinical Endocrinology (AACE) [84–86] further broadened access, transitioning CGM from a specialised technology for select subgroups into the gold standard of routine clinical care.

### Current landscape of CGM in early-stage type 1 diabetes

**Fig. 2:**
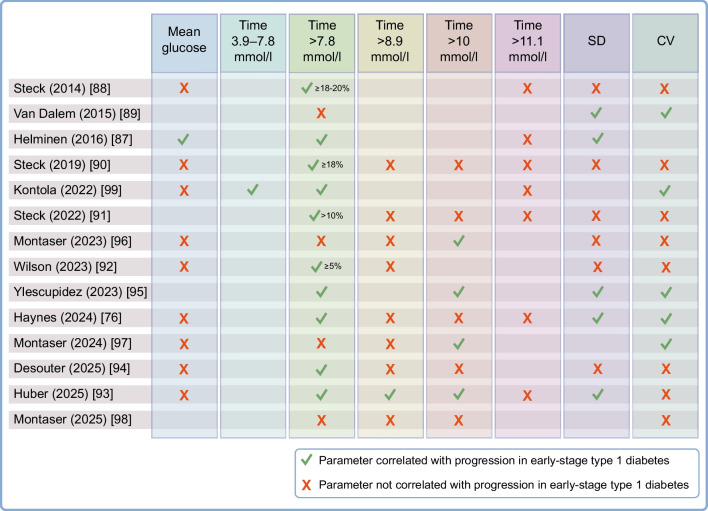
CGM metrics associated with progression in early-stage type 1 diabetes. Green ticks indicate metrics significantly correlated with progression in early-stage type 1 diabetes, while red crosses indicate no significant association. Blanks represent where information was not reported. T1D, type 1 diabetes. This figure is available as part of a downloadable slideset

To identify relevant studies examining CGM in early-stage type 1 diabetes, we conducted a targeted narrative search of MEDLINE via PubMed (January 2000–September 2025) using the terms ‘continuous glucose monitoring’, ‘CGM’, ‘screening’, ‘autoantibodies’ and ‘progression’. Citation searching was also performed. Inclusion criteria were (1) studies using CGM in individuals with islet autoantibodies, stage 1 or stage 2 type 1 diabetes; (2) studies reporting glycaemic metrics or progression endpoints; and (3) studies published in English. Exclusion criteria were studies in established type 1 diabetes, type 2 diabetes or maturity-onset diabetes of the young, or those lacking CGM-derived metrics. We identified 14 studies that have examined CGM in early-stage type 1 diabetes (Table 1), primarily using cross-sectional comparisons (autoantibody status or stage) or progression endpoints to stage 3 and often focusing on thresholds for time above specific glucose cut-offs (Fig. 2).

**Table 1:** Summary of studies evaluating CGM to stage and predict progression in early-stage type 1 diabetes (*n*=14)

Study^a^	Population (antibody status, age [years])	CGM model and wear duration^b^	Key CGM metrics measured	Key outcomes
Steck (2014) [88]	*N*=23 ≥2 Aab (*n*=14), age 13.8 ± 3.5 0 Aab (*n*=9), age 13.6 ± 2	Dexcom SEVEN 5–7 days	Mean glucose, max. glucose, SD, CV, TA7.8 mmol/l, TA11.1 mmol/l, AUC	≥18–20% TA (≥) 7.8 mmol/l could be used to predict progression to T1D in Aab-positive children: TA (≥) 7.8 mmol/l: 31.0 ± 18.0% in progressors (*n*=5) vs 12.0 ± 7.0% in non-progressors (*n*=9), *p*=0.04
Van Dalem (2015) [89]	*N*=51 ≥2 Aab (*n*=22), median (range) age 19 (12–41) 0 Aab (*n*=20), median (range) age 18 (12–40) Stage 3 T1D (*n*=9), median (range) age 20 (13–36)	Medtronic iPro2 5 days	SD, CV, IQR, TA7.8 mmol/l	CGM measurements (SDday, IQRday and CVday) above the range of those from healthy control individuals had higher diagnostic efficiency for detecting or predicting dysglycaemia than elevated SMBG variables (Rangeday and SDday) (77–82% vs 73%) Those who developed IGT or diabetes during follow-up (*n*=5) had higher GV variables than those who remained euglycaemic (*n*=12)
Helminen (2016) [87]	*N*=20 ≥2 Aab (*n*=10), age 9.8 ± 4.1 0 Aab (*n*=10), age 9.9 ± 4.5	Dexcom G4 Platinum 7 days	Mean glucose, max. glucose, range, SD, TA7.8 mmol/l, TA11.1 mmol/l, AUC, MAGE	Aab-positive individuals have higher average glucose and glucose variability than matched Aab-negative control individuals: Glucose (mmol/l): 5.4 ± 0.6 vs 4.7 ± 0.3, *p*=0.018 SD (mmol/l): 1.2 ± 0.5 vs 0.8 ± 0.2, *p*=0.04 TA (≥) 7.8 mmol/l: 5.9 ± 7.0% vs 0.4 ± 0.4%, *p*=0.04
Steck (2019) [90]	*N*=23 ≥2 Aab (*n*=23), age 13.9 ± 3.8 progressors, 16.6 ± 4.5 non-progressors	Dexcom SEVEN Plus or Dexcom G4 Platinum 5–7 days (on multiple occasions)	Mean glucose, max. glucose, SD, CV, range, TA6.7 mmol/l, TA7.8 mmol/l, TA8.9 mmol/l, TA10.0 mmol/l, TA11.1 mmol/l, AUC	≥18% TA (≥) 7.8 mmol/l predicts progression to T1D in Aab-positive children: TA (≥) 7.8 mmol/l: 24.0 ± 17.0% in progressors (*n*=8) vs 8.0 ± 6.0% in non-progressors (*n*=15), *p*=0.005
Kontola (2022) [99]	*N*=46 0 Aab (*n*=9), median (range) age 9.3 (3.9–15.2) 1 Aab (*n*=6), median (range) age 12.3 (7.4–13.1) ≥2 Aab (*n*=31), median (range) age: stage 1 (*n*=12), 9.7 (5.1–25.4); stage 2 (*n*=11), 15.1 (4–20.2); stage 3 (*n*=8), 9.7 (4.5–19.7)	Dexcom G6 10 days	Mean glucose, CV, MAGE mean, HBGI mean, CGM estimated HbA_1c_, TA7.8 mmol/l, TA11.1 mmol/l, TA13.9 mmol/l, TB3.9 mmol/l, TB3.0 mmol/l, TIR3.9–7.8 mmol/l, AUC, OGTT metrics	CGM can identify asymptomatic individuals in stage 3 T1D and dysglycaemic individuals in stage 2 T1D prior to an OGTT: TA (≥) 7.8 mmol/l: median (IQR) 3.95% (2.21–6.19) (0 Aab) vs 5.95% (3.6–10.3) (stage 1) vs 44.5% (17.0–43.2) (stage 3), *p*<0.01 TIR3.9–7.8 mmol/l: 94.0 ± 2.7% (0 Aab) vs 90.9 ± 4.9% (stage 1) vs 67.8 ± 13.4% (stage 3), *p*<0.001 CV: 15.5 ± 1.8% (0 Aab) vs 19.8 ± 1.9% (stage 1) vs 30.8 ± 6.5% (stage 3), *p*<0.001
Steck (2022) [91]	*N*=91 ≥1 Aab (*n*=91), median (IQR) age 10.5 (6.8–13.2) progressors, 11.7 (8.1–14.5) non-progressors	Dexcom G4 Platinum or Dexcom G6 7–10 days, (every 6 months)	Median glucose, SD, CV, range, TA6.7 mmol/l, TA7.8 mmol/l, TA8.9 mmol/l, TA10.0 mmol/l, TA11.1 mmol/l, MAGE, MODD, AUC	>10% TA (>) 7.8 mmol/l is associated with high risk of progression to stage 3 T1D in Aab-positive individuals: TA (≥) 7.8 mmol/l: median (IQR) 21.0% (13.0–33.0) in progressors (*n*=16) vs 3.0% (1.0–7.0) in non-progressors (*n*=75), *p*<0.0001
Montaser (2023) [96]	*N*=60 0 Aab (*n=*21), age 27 ± 9.9 1 Aab (*n=*18), age 23.5 ± 11.9 ≥2 Aab (*n=*21), age 20.7 ± 10.2	Dexcom G4 Platinum 7 days	Mean glucose, SD, CV, TA7.8 mmol/l, TA8.9 mmol/l, TA10.0 mmol/l, TB3.9 mmol/l, TB3.0 mmol/l, LBGI, HBGI, ADRR, post-SLMM CGM metrics	Three metrics differed between groups: TA (>) 10.0 mmol/l, *p*=0.040 (negative vs 1 Aab *p* = 0.352, negative vs ≥2 Aab, *p *= 0.012, 1 Aab vs ≥2 Aab, *p*= 0.144) Overnight CGM incremental AUC, *p*=0.005 (negative vs 1 Aab *p* = 0.012, negative vs ≥2 Aab *p* = 0.005, 1 Aab vs ≥2 Aab *p* = 0.012) TA (>) 10.0 mmol/l for 75 min post standard liquid meal, *p*=0.004 (negative vs 1 Aab *p *= 1.000, negative vs ≥2 Aab *p*= 0.012, 1 Aab vs ≥2 Aab *p*= 0.018) TA (>) 7.8 mmol/l was not as predictive as TA (>) 10.0 mmol/l
Wilson (2023) [92]	*N*=105 0 Aab (*n=*10), median (IQR) age 16.3 (15.1–18) ≥2 Aab (*n*=95): median (IQR) age: stage 1 (*n*=53), 17.2 (11.7–36.4); stage 2 (*n*=42), 15.5 (11.6–37.5)	Dexcom G4 Platinum 7 days	Mean glucose, SD, CV, max. glucose, min. glucose, range, TA6.7 mmol/l, TA7.8 mmol/l, TA8.9 mmol/l, CONGA, DySF, MAGE, MODD	≥5% TA (≥) 7.8 mmol/l was predictive of progression to stage 3 T1D in Aab-positive individuals: TA (≥) 7.8 mmol/l: 8.0 ± 8.0% in progressors (*n*=29) vs 4.7 ± 6.0% in non-progressors (*n*=66), *p*=0.03
Ylescupidez (2023) [95]	*N*=93 ≥2 Aab (*N*=93), stage 1 (*n=*58), stage 2 (*n*=35); age 18.2 ± 13.3 progressors, 26.6 ± 15.2 non-progressors	Dexcom G4 Platinum 7 days (three occasions, 6 months apart)	CV, SD, range, TA7.8 mmol/l, TA11.1 mmol/l, time 3.3–7.8 mmol/l, ADRR, CONGA, GRADE, HBGI, MAGE, MODD, max. glucose	4/7 OGTT metrics and 29/48 CGM metrics were statistically different between Aab-positive progressors and non-progressors including: TA (>) 7.8 mmol/l: median (IQR) 5.45% (2.16–11.02) in progressors (*n*=34) vs 2.46% (0.7–6.45) in non-progressors (*n*=59), *p*=0.026 Individual CGM metrics did not exceed an AUC of 70% for prediction of T1D. Combining CGM metrics improved the adjusted ROC AUC for prediction of subsequent T1D to 76.6% Index60 and DPTRS had the highest unadjusted ROC AUC for prediction of subsequent T1D
Haynes (2024) [76]	*N*=55 0 Aab (*n*=24), age 4.7 ± 1.9 ≥2 Aab (*n*=31), age 4.4 ± 1.8	Dexcom G6 14 days (consecutive days, with two consecutive sensors)	Mean glucose, CV, SD, TA7.8 mmol/l, TA8.9 mmol/l, TA10.0 mmol/l, TA11.1 mmol/l, TB3.9 mmol/l, TB3.5 mmol/l, TB3.0 mmol/l, MAGE, CONGA	Very young Aab-positive children have higher SD, CV and TA (>) 7.8 mmol/l than Aab-negative children: SD (mmol/l): median (IQR) 1.1 (0.9–1.3) vs 0.9 (0.8–1.0), *p*<0.001 CV: 17.3% (16.0–20.9) vs 14.7% (12.9–16.6), *p*<0.001 TA (>) 7.8 mmol/l: 8.0% (4.4–13.0) vs 3.3% (1.4–5.3), *p*=0.005
Montaser (2024) [97]	*N*=42 0 Aab (*n*=21), age 27 ± 9.9 ≥2 Aab (*n*=21), age 20.7 ± 10.2	Dexcom G4 Platinum 7 days	Mean glucose, CV, range, IQR, TA6.7 mmol/l, TA7.8 mmol/l, TA8.9 mmol/l, TA10.0 mmol/l, TB3.9 mmol/l, TB3.0 mmol/l	Three metrics differed between groups: CV, *p*=0.028 Range, *p*= 0.035 TA (>)10.0 mmol/l, *p*= 0.04 In a classifier model, CV, range, TA8.9 mmol/l, Gmax and IQR were the most significant features capturing variability and dysglycaemia in the two risk groups
Desouter (2025) [94]	*N*=34 ≥2 Aab (*N*=34), median (IQR) age 16.6 (13.4–23.4)	Medtronic iPro2 5 days (semi-annually)	Mean glucose, SD, CV, IQR, TA6.7 mmol/l, TA7.8 mmol/l, TA8.9 mmol/l, TA10.0 mmol/l, AUC glucose	Considerable inter- and intra-variation TA6.7 mmol/l and TA7.8 mmol/l increased in progressors (*n*=17) within the last 3 years before diagnosis compared with most non-progressors (*n*=17) In a univariate prediction model TA (≥) 6.7 mmol/l was the most effective CGM metric (AICc=75) at predicting progression to stage 3 T1D OGTT-derived AUC glucose outperformed CGM TA (≥) 6.7 mmol/l (AICc=71.1). A multivariable CGM model of mean glucose and IQR achieved a similar AIC (AICc=72.5)
Huber (2025) [93]	*N*=97 0 Aab (*n*=33), median (IQR) age 11 (10–13) ≥2 Aab (*n*=64), median (IQR) age: stage 1 (*n*=46), 9 (7–11), stage 2 (*n*=18), 9.5 (7.3–11.8)	Dexcom G6 10 days (multiple occasions)	Mean glucose, SD, CV, max. glucose, TA7.8 mmol/l, TA8.9 mmol/l, TA10.0 mmol/l, TA11.1 mmol/l, TB5.6 mmol/l, TB4.4 mmol/l	Several CGM parameters were significantly different between Aab-negative control individuals and those with stage 1 and stage 2 T1D, including: TA (>) 7.8 mmol/l: stage 1: median (IQR) 11.3% (5.5–26.9); stage 2: 31% (16.1,44.6); control: 6.6% (3.2–16.2%) (control vs stage 1 *p*= 0.04, control vs stage 3 *p*= 0.0003, stage 1 vs stage 2 *p*= 0.01) A composite progression score using SD, TA (>) 7.8 mmol/l, TA (>) 8.9 mmol/l and TA (>) 10.0 mmol/l could identify those who progressed to stage 3 (*n*=11) (AUC 0.88 [95% CI 0.77, 0.99], *p*<0.0001) TA (>) 7.8 mmol/l ≥10% had a very low specificity and was associated with a low risk for stage 3 type 1 diabetes
Montaser (2025) [98]	*N*=39 1 Aab (*n*=18), age 23.5 ± 11.9 ≥2 Aab (*n*=21), age 20.7 ± 10.2	Dexcom G4 Platinum 7 days	CV, TA7.8 mmol/l, TA8.9 mmol/l, TA10.0 mmol/l, AUC glucose	Post standardised liquid mixed meal TA (>) 10.0 mmol/l differed between groups (*p*=0.020) A model combining T1D genetic risk score and incremental AUC glucose differentiated groups with ROC AUC of 0.93 (95% CI 0.83, 1.00)

Values are mean ± SD unless otherwise stated

^a^Studies listed were identified through a targeted narrative search of MEDLINE via PubMed (January 2000–September 2025)

^b^CGM was blinded in all studies except for that by Kontola et al [99], in which CGM was unblinded on request

Aab, autoantibody; AICc, corrected Akaike information criterion; CONGA, Continuous Overall Net Glycaemic Action; DySF, Dynamic Stress Factor; GRADE, Glycaemic Risk Assessment Diabetes Equation; Gmax, maximal glucose amplitude; HBGI, High Blood Glucose Index; IGT, impaired glucose tolerance; LBGI, Low Blood Glucose Index; MAGE, mean amplitude of glycaemic excursion; MODD, mean of daily differences; ROC, receiver operating characteristic; SLMM, standardised liquid mixed meal; T1D, type 1 diabetes; TA, % time above (specified as ≥ or > in results depending on the study); TB, % time below; TIR, time in range

Early small studies demonstrated that autoantibody-positive individuals had higher mean glucose levels, glycaemic variability and time above 7.8 mmol/l glucose than autoantibody-negative control individuals [87], both as a group and among those who progressed to stage 3 [88, 89]. Steck et al were the first to propose a predictive threshold, identifying that spending ≥18–20% time above 7.8 mmol/l glucose signalled an increased risk of progression to stage 3 type 1 diabetes [88].

Subsequent larger cohorts have refined these thresholds: ≥18% time ≥7.8 mmol/l glucose predicted progression in children with two or more autoantibodies [90], the Autoimmunity Screening for Kids (ASK) cohort demonstrated that >10% time spent above 7.8 mmol/l glucose was strongly predictive of progression to stage 3 within a year in individuals with one or more autoantibody [91], and the TrialNet Pathway to Prevention (TNPTP) cohort reported that a value as little as ≥5% of time spent at ≥7.8 mmol/l glucose was predictive of progression to stage 3 in autoantibody-positive individuals [92]. A recent study, however, demonstrated that a threshold of ≥10% of time above 7.8 mmol/l glucose had low specificity and was associated with limited risk for stage 3 type 1 diabetes, particularly in those with stage 1 disease, while a composite progression score combining SD with time above 7.8, 8.9 and 10.0 mmol/l glucose achieved superior discrimination than any single metric (AUC 0.88) [93]. A small study from the Belgian diabetes registry identified that time above 6.7 mmol/l glucose was the most effective univariate CGM predictor of progression, although the OGTT-derived AUC glucose outperformed CGM in univariable models; combining CGM features with HbA_1c_ improved performance [94].

In a study by Ylescupidez et al, while 29 of 48 daytime CGM-derived metrics were found to significantly differ between progressors and non-progressors, the predictive utility of individual measures, and even of combined panels including CV, mean glucose, IQR, SDwsh (within-participant average hourly SD) and hypo-/hyperglycaemic index, remained modest and consistently inferior to OGTT-based indices such as Index60 and DPTRS [95]. However, combining CGM-derived features with participant characteristics or standardised liquid meal protocols improves discrimination, with incremental AUCs and postprandial percentage time spent at >10.0 mmol/l glucose distinguishing antibody-negative from antibody-positive groups, and integration with genetic risk scores achieving ROC AUCs up to 0.93 [96–98].

Cross-sectional analyses have consistently shown graded increases in glycaemic variability (SD, CV, mean amplitude of glycaemic excursion [MAGE]) and time above 7.8, 8.9 and 10.0 mmol/l glucose across control, stage 1 and stage 2 individuals [76, 93, 99]. Very young children (mean age ~4 years) with multiple autoantibodies also displayed higher variability and increased time above 7.8 mmol/l than antibody-negative control children (SD 1.1 vs 0.9 mmol/l, CV 17.3% vs 14.7%, time >7.8 mmol/l 8.0% vs 3.3%) [76]. In some settings, thresholds >10.0 mmol/l were more discriminative than those >7.8 mmol/l, particularly postprandially [96].

Across studies, time above 7.8 mmol/l glucose consistently signals higher risk, although thresholds vary from as little as 5% to as high as 20% (Table 1, Fig. 2). Nonetheless, recent recommendations have formalised the use of 7.8 mmol/l glucose as a staging threshold, defining stage 2 as ≥10% and stage 3 as ≥20% of time above this level, confirmed by additional non-CGM glucose measures [13]. A recent meta-analysis highlighted that higher CGM cut-offs increase specificity at the expense of sensitivity, possibly missing some high-risk individuals, and that predictive performance is strengthened when metrics are interpreted alongside factors such as sex, family history and autoantibody profile [100].

### CGM as a tool to guide initiation of insulin or immunotherapy

Current consensus is lacking on the use of CGM to guide insulin initiation in early-stage type 1 diabetes, although recent recommendations support a ‘treat-to-target’ approach [101]. However, early use of insulin carries risks, given the absence of defined dosing strategies, with potential for hypoglycaemia and uncertain long-term adherence before overt symptoms appear.

The role of CGM in monitoring responses following immunotherapy is also currently undefined, but clinical trials increasingly include CGM metrics as secondary endpoints. CGM can detect early hyperglycaemic patterns that may reflect declining beta cell function, but treatment effects on C-peptide do not always translate into short-term CGM improvements, as seen in the INNODIA MELD-ATG trial, where low-dose antithymocyte globulin preserved stimulated C-peptide but did not improve CGM-derived time in range at 12 months [102].

### Potential pitfalls for CGM use in early-stage type 1 diabetes

#### Heterogeneity

Heterogeneous study designs may reduce the pre-test probability of progression in some analyses [91, 94]. Several of the studies to date compared multiple antibody-positive individuals with antibody-negative individuals [76, 87–89, 92, 93, 97] while others compared any individual with one or more antibody with antibody-negative individuals [91, 94]. Some studies focused on progression to stage 3 type 1 diabetes in individuals with multiple autoantibodies [90, 92, 95], while others relied on cross-sectional data to distinguish groups by antibody status or type 1 diabetes stage [87, 88, 93, 99]. Further inconsistency arises from thresholds reported as ‘>7.8 mmol/l’ vs ‘≥7.8 mmol/l’, a small distinction that nonetheless alters classification between cohorts. Notably, age-related discrepancies have been observed in normoglycaemic individuals without diabetes. While time above 7.8 mmol/l glucose is typically low in younger adults [103], a recent larger analysis of an older population demonstrated that ~10% of time spent above 7.8 mmol/l glucose is expected, even when stratified to include only those under 60 years of age and without obesity (BMI <30 kg/m^2^) [104]. Differences in glycaemic profiles in the population without diabetes have been shown to change with age [105, 106]. This likely reflects physiological changes associated with ageing [107], such as declining insulin sensitivity, altered postprandial responses and reduced physical activity levels. This underscores the need for caution when applying uniform glycaemic thresholds across age groups, and hence age-specific thresholds may be necessary when using CGM for staging within adult screening programmes, in order to avoid false-positive findings. Despite the availability of data from large, normative studies [103, 104, 108], there is currently no consensus on what constitutes a ‘normal’ CGM profile [19].

#### Study numbers

The small number of studies to date using CGM in early-stage type 1 diabetes have resulted in around 779 sets of CGM data (Table 1). In part this is due to CGM being a relatively new tool, with the US Food and Drug Administration (FDA) approving the first commercially available device in 1999 [109]. Further, there are limited data on adult populations with early-stage disease, with few studies having a mean age >18 years [96, 97].

#### Sensor types

Four studies to date used a Dexcom G6 or newer sensor, whereas the remaining ten studies used older generation devices (Dexcom G4, Medtronic iPro 2 and Dexcom SEVEN) (Table 1). It is likely that device-dependent differences play an important role in the heterogeneity observed. However, such differences are not confined to older models [110, 111]. A recent comparative study demonstrated that even current generation systems can diverge substantially, resulting in mean glucose differing by up to 1.1 mmol/l and time in range (3.9–7.8 mmol/l) varying by nearly 20% [112]. Such discrepancies highlight that CGM-derived metrics are not interchangeable across devices [113, 114]. This variability in part reflects inconsistency in comparator methods in sensor development and calibration, with capillary vs venous plasma sampling producing ranging biases depending on the measurement method [115–117], contributing to more than 5% bias existing between individual devices of the same brand [117–119]. This has motivated calls to action to standardise comparator type, sampling frequency and analytical performance to ensure comparability [116, 119, 120], which we support.

#### Surrogate for beta cell health

CGM does not directly measure beta cell health, which is better assessed through metabolic tests and modelling [39]. This underscores a broader challenge: glycaemia-based tools may be highly sensitive but less specific indicators of underlying pathophysiology. Glucose represents a surrogate of beta cell health, and CGM adds a further layer of indirection by monitoring interstitial rather than plasma glucose. CGM offers a practical method for detecting glucose abnormalities but cannot disentangle whether observed dysglycaemia arises from defects in insulin secretion, sensitivity or both. CGM abnormalities near disease onset have been shown to parallel changes in OGTT-derived variables, although both exhibit substantial intra- and interindividual variability [94]. As such, OGTTs (including derived metrics) and CGM offer distinct yet complementary windows into beta cell health, underscoring the rationale for evaluating CGM in early-stage type 1 diabetes as either an adjunct or a pragmatic alternative to periodic metabolic tests. However, its role in staging and predicting disease or serving as a surrogate endpoint remains to be fully defined [93]. As data accumulate, novel CGM analyses may help stratify risk and define subgroups with divergent progression trajectories [97, 98, 100].

## Practical considerations for CGM use in early-stage type 1 diabetes

### Psychological impact and acceptability to the general public

Receiving a positive autoantibody result has been shown to cause significant initial distress, particularly for mothers of children receiving this [121–123]. The use of CGM as a method of quantifying risk could provide some reassurance due to the unpredictable nature of progression to stage 3 type 1 diabetes. A single study evaluating the acceptability of CGM in a cohort from the ENDIA study found that parents had largely positive experiences of using CGM, and felt that having additional knowledge about their child’s glucose patterns helped them further understand the risk of progression to stage 3 type 1 diabetes [124].

However, the possibility of heightened anxiety, as is seen in established type 1 diabetes [125] due to increased visibility of glucose levels, needs better understanding [126]. Across multiple studies, perceived burdens of CGM include alarms and the volume and complexity of CGM data [126–128]. Additionally, pain and reactions at insertion sites are notable in type 1 diabetes [129], which has been reflected in concerns raised in studies of CGM in early-stage type 1 diabetes [19, 124]. This requires further study, including across age groups.

### Blinded vs unblinded use

Positive autoantibody status alone has been associated with behaviour changes, including changes to meal planning, and increased blood glucose monitoring [130, 131], which may be amplified with access to real-time data. It is therefore recommended in consensus guidelines that CGM should ideally be blinded for diagnostic and prognostic purposes [19] to minimise the influence of physiological and behavioural factors, such as meal timing, physical activity and stress. The study by Kontola et al is the only study to date that has provided unblinded CGM [99], with 35% of participants or their guardians requesting access to unblinded glucose data (median age of participants 11.7 years [range 3.9–25.4]). While unblinded systems can empower individuals and provide transparency [132], in this setting appropriate education would be necessary [19]. Given Kontola et al’s experience [99] one might consider a hybrid approach, for example offering unblinding after a defined observation period. However, more studies are needed that compare blinded vs unblinded use in early-stage type 1 diabetes.

### Duration and frequency of CGM wear

The optimal duration and frequency of CGM wear in early-stage type 1 diabetes remains under consideration. In established type 1 diabetes, multiple studies suggest that 12–15 days of data [133–135] are sufficient to provide reliable estimates of mean glucose, glycaemic variability and time in range, with strong correlations with 3 month glycaemic profiles [136–139]. This may be an appropriate target for the duration of CGM wear in early-stage type 1 diabetes in the context of prediction. The frequency of CGM use in early-stage type 1 diabetes must balance adequate data capture with participant burden and cost, arising from the device itself, the need for training and educational resources, and data interpretation. Current recommendations suggest repeat CGM screening every 6–36 months in adults, depending on the immune burden and risk of progression [13, 138, 140]. In children, frequency varies by age and immune burden, ranging from every 3 months in those aged under 3 years or with dysglycaemia to annually in those aged over 9 years in stage 1 [13, 19, 91]. However, considering the relatively common metabolic fluctuations in early-stage type 1 diabetes, prolonged CGM wear may be warranted in selected individuals to capture evolving glycaemic patterns; this requires further study.

Another important consideration is data quality. International consensus guidelines recommend ≥70% CGM wear time in established type 1 diabetes to ensure reliable metrics [137, 138] and that all CGM data should be analysed to avoid the introduction of bias [138]. However, these standards need further elucidation in early-stage type 1 diabetes.

### Reliability

Although the accuracy of CGM devices has improved, even current generation devices show inter-device variability that affects derived metrics [110–112, 115, 119, 141]. These discrepancies reflect variations in calibration methods and signal processing, compounded by the absence of a universally standardised reference for factory calibration, underscoring the need for harmonised validation protocols across devices [116, 119, 120]. The current FDA approach for integrated CGM (iCGM) systems [142] defines device accuracy criteria for regulatory approval but does not standardise study design or comparator methodology; hence, devices may comply with special control requirements, yet differ in performance [114, 119, 143].

Beyond inter-device variability arising from systematic differences between manufacturers, models, and sensors of the same model, reliability is also shaped by data completeness and user acceptability; attrition due to incomplete data or participant refusal can limit both trial feasibility and real-world adoption. Biological and technical factors add further variability and indeed can result in deviations from the ‘true’ value over the period of wear. Local blood flow, hydration status and inflammatory responses at the insertion site can alter tissue fluid dynamics, while most sensors, relying on glucose oxidase-based electrochemistry, are susceptible to interference from other molecules. Depending on enzyme formulation and applied voltage, endogenous metabolites such as uric acid and ascorbic acid, as well as exogenous compounds including paracetamol (acetaminophen) and dietary components, can perturb current flow at the sensor tip and artificially shift reported glucose values [144]. User surveys also highlight perceived accuracy limitations under real-world conditions, including during dehydration or illness or with use of over-the-counter medications and vitamin C, although formal validation studies remain sparse for many of these scenarios [145]. It remains unclear if current CGM systems experience reduced accuracy across a broad range of physiological conditions; however, recent consensus statements suggest that modern CGM systems retain acceptable accuracy in people with advanced chronic kidney disease and on dialysis [146].

Finally, interstitial-to-plasma glucose lag remains a consideration. Although less likely to cause systematic misclassification in early-stage research, where analyses are retrospective and abnormalities modest, it remains a source of noise [147, 148].

Collectively, although individually small, these biological, behavioural and sensor related-sources of variability can accumulate, potentially influencing prognostic/diagnostic thresholds used in early-stage type 1 diabetes, and reinforce the need for rigorous standardisation before CGM metrics can be reliably accepted as regulatory endpoints.

### Cost and access

In established type 1 diabetes, CGM has been shown to be cost-effective compared with SMBG, through reductions in healthcare use, hospitalisations and diabetes complications [149, 150].

In early-stage type 1 diabetes, the cost-effectiveness of CGM is unknown. One can speculate that CGM might offset screening and monitoring costs by reducing reliance on resource-intensive tests such as the OGTT or by enabling earlier, risk-stratified interventions; however, formal economic assessments are required. These should ideally model multiple scenarios using real-world screening data and healthcare use rates to assess the value of CGM in early-stage type 1 diabetes.

### Analysis of CGM data

Analytical approaches to CGM in early-stage type 1 diabetes are evolving. Most studies use simple descriptive metrics (e.g. percentage time above, below or within ranges), but such static summaries lose the richness of glucose as a continuous time series and are sensitive to missing or irregular data [151]. This has prompted calls for more advanced methods, including functional data analysis, machine learning and artificial intelligence [152], that could offer deeper insights into glucose dynamics.

Some emerging approaches show potential in early-stage type 1 diabetes. Glucodensity [153], which models glucose as a distributional function rather than discrete values, has uncovered risk phenotypes across normoglycaemic, type 2 diabetes and type 1 diabetes cohorts that are invisible to conventional metrics [153, 154]. Machine learning algorithms trained on 16 point OGTT curves have been applied to CGM data during standardised OGTTs, accurately identifying insulin resistance and beta cell dysfunction in a simple, at-home testing framework [155]. A similar testing framework has also been investigated in early-stage type 1 diabetes [97] using risk classifiers based on CGM summary features during a 2 h OGTT. While informative, this approach still compresses CGM data into static snapshots and may not fully capture important temporal dynamics describing underlying physiology.

Time series decomposition and forecasting approaches are also gaining traction, with recent work in type 2 diabetes showing that decomposed CGM traces can cluster representative glucose profiles and predict 6 month therapeutic responses [156], illustrating CGM’s emerging potential as a predictive biomarker. Adapting these approaches could enhance CGM’s prognostic value in early-stage type 1 diabetes by anticipating progression and detecting subtle metabolic inflections. As immunomodulatory trials expand, these approaches may eventually also inform the prediction of treatment response.

Furthermore, a recent FDA commentary underscores that, as CGM-derived endpoints gain prominence in regulatory decision-making, CGM data pose distinct statistical challenges, including epoch-level irregularities, data anomalies and non-random missingness, that must be handled transparently and consistently in trial analysis plans [157].

### Burden of monitoring and importance of acceptability in children

Beyond the technical and clinical considerations, the lived experiences of children and families must be recognised. Many children with stage 1 or 2 type 1 diabetes do not require intervention for years, making repeated clinical visits difficult to justify. Population-based screening will require follow-up strategies that prioritise acceptability, practicality and sustainability. Clear communication and education are essential to prevent disengagement. By offering a less burdensome option, CGM may improve adherence to and retention in longitudinal monitoring, especially when paired with psychosocial support and education [124, 126].

## Regulatory perspectives

The FDA and European Medicines Agency (EMA) have acknowledged the clinical utility of CGM in the management of established type 1 diabetes, approving multiple devices to optimise glycaemic control and reduce hypoglycaemia risk. Their most recent guidance recognises CGM as a valuable tool but does not elevate it to a primary endpoint in prevention or preservation trials. The FDA’s 2023 draft guidance [158] maintains HbA_1c_ as the primary endpoint, accepts CGM-based hypoglycaemic measures as validated, and treats other metrics such as time in range as exploratory. Similarly, the EMA’s 2023 final guideline on diabetes trials [159, 160] encourages CGM use and explicitly accepts 24 h glucose profiles as secondary endpoints, but continues to require diabetes incidence (stages 1–2) or C-peptide/HbA_1c_ (stage 3) as primary endpoints. Neither agency has yet established a pathway for CGM as a primary endpoint in early-stage type 1 diabetes.

Moving towards such acceptance will require alignment with biomarker qualification frameworks such as FDA’s BEST (Biomarkers, EndpointS, and other Tools) [18] and the EMA’s process for novel biomarker validation, which emphasises analytical validity, clinical relevance and a defined context of use [161]. Recent FDA approvals of the Dexcom Stelo and Abbott’s Rio and Lingo for adults who do not use insulin [162–164] underscore a regulatory openness to broader CGM applications, highlighting the potential relevance of CGM in early-stage type 1 diabetes.

To date, most evidence supports CGM’s role as a prognostic tool. Its potential as a predictive biomarker, identifying subgroups most likely to benefit from immunotherapy or beta cell preservation strategies, remains to be explored*.* This distinction is critical for regulatory qualification and for defining the context of use in future implementation.

Looking ahead, prospective studies will be essential to establish CGM metrics as prognostic biomarkers for disease progression and supportive endpoints in intervention trials, particularly for patient selection, stratification and enrichment contexts where most successful biomarker qualifications have occurred [161]. Clear research priorities must be defined: Short-term efforts should focus on harmonising comparator methods and device validation; mid-term efforts on generating robust prospective evidence for CGM metrics as prognostic biomarkers; and long-term efforts on integration into regulatory biomarker qualification pathways (Table 2).

**Table 2:** Research priorities in CGM for early-stage type 1 diabetes

Duration	Priority
Short term	• Determine and agree internationally harmonised standards for CGM evaluation protocols between regulatory authorities and industry stakeholders to reduce inter- and intra-sensor variability • Define normative thresholds (age-specific) in antibody-negative control population and accompanying inter- and intra-sensor variability to inform CGM thresholds for staging and prediction • Determine acceptability of CGM wear in the early-stage type 1 diabetes general population compared with other metrics used in staging and monitoring (e.g. HbA_1c_, OGTT, venous glucose) • Define the optimal frequency and duration of repeat CGM wear to monitor or predict progression • Define the optimal type 1 diabetes stage(s) for CGM use to predict progression • Quantify the impact of behaviour change with unblinded and blinded CGM wear and subsequent impact on staging and prediction of progression
Medium term	• Develop specific educational tools and guidance for clinicians and CGM users about the role of CGM in early-stage type 1 diabetes • Define the role of CGM in staging type 1 diabetes and as a surrogate prognostic biomarker for disease progression singularly and with other markers of beta cell health • Define the role of CGM in guiding the initiation of, and responses following, immunotherapy and/or insulin therapy • Evaluate the cost-effectiveness of CGM as an alternative to the gold standard measure (OGTT)
Long term	• Establish large general population CGM datasets for early-stage type 1 diabetes (children and adults) • Define the role of functional analysis and machine learning as alternative techniques to analyse CGM data in early-stage type 1 diabetes • Determine the long-term acceptability of CGM wear in early-stage type 1 diabetes individuals and develop support frameworks • Understand the factors that influence CGM uptake for equitable access to CGM in early-stage type 1 diabetes • Integrate CGM into regulatory biomarker qualification pathways for use as an endpoint in primary and secondary prevention trials

## Conclusion

**Fig. 3:**
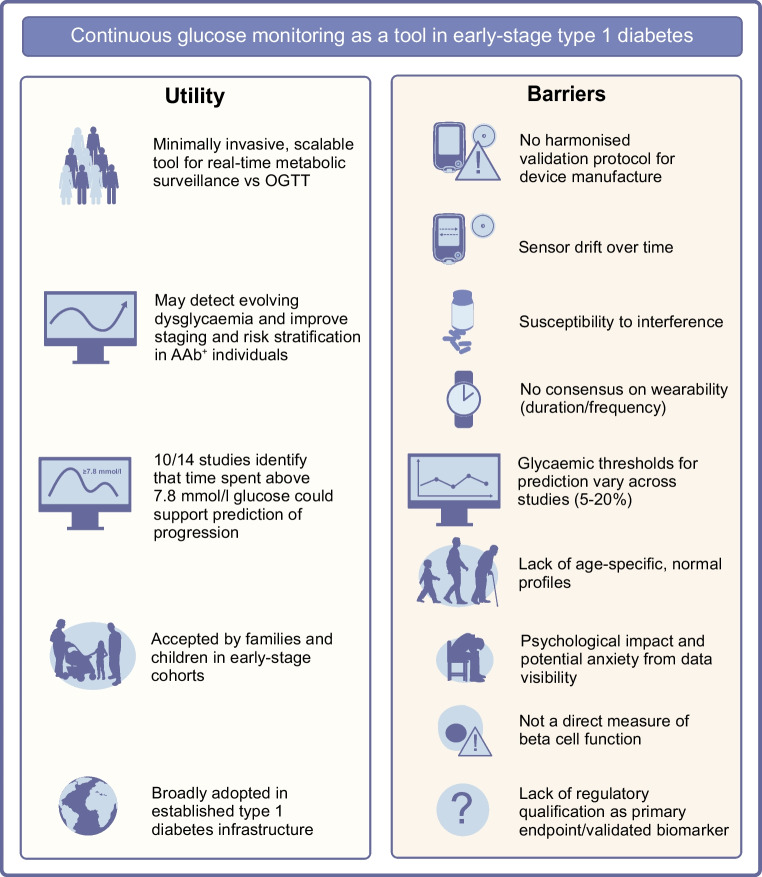
Schematic overview of the potential utility of and key barriers to implementing CGM in early-stage type 1 diabetes. This figure is available as part of a downloadable slideset

CGM has the potential to enhance assessment in early-stage type 1 diabetes, addressing critical unmet needs by providing minimally invasive, real-time assessments of glucose dynamics and detecting subtle, transient dysglycaemia that may be missed by intermittent testing. These features position CGM as a valuable complement or, in some contexts, a pragmatic alternative to the less practical gold standard OGTT. However, despite this promise, important barriers must be addressed. These include the need for appropriate contextualisation of comparative normal values, substantial inter-sensor variability (systematic differences between manufacturers, models, and sensors of the same model) and intra-sensor variability causing imprecision, bias and sensor drift over the wear period (arising from insertion effects, tissue changes, enzyme degradation or physiological conditions), uncertainty about optimal wear duration and frequency, lack of international standardisation, potential psychological burdens and unknown cost-effectiveness (Fig. 3).

Establishing CGM as a reliable tool for screening, staging and management in early-stage type 1 diabetes will require rigorous methodological validation, particularly if CGM-derived metrics are to be considered as prognostic biomarkers or trial endpoints. Prospective studies must define clinical validity, optimal implementation strategies and cost-effectiveness in early-stage disease. We therefore call for coordinated research efforts across academic, clinical, industry and regulatory partners to support the standardisation, integration and eventual regulatory qualification of CGM within early-stage type 1 diabetes frameworks.

## Supplementary Information

Below is the link to the electronic supplementary material.

Slideset of figures (PPTX 326 KB)

## Abbreviations

CGM: Continuous glucose monitoring
DKA: Diabetic ketoacidosis
DPTRS: Diabetes Prevention Trial–Type 1 Risk Score
EMA: European Medicines Agency
FDA: Food and Drug Administration
IVGTT: Intravenous glucose tolerance test
MMTT: Mixed-meal tolerance test
OGTT: Oral glucose tolerance test
SMBG: Self-monitoring of blood glucose

## Authors’ relationships and activities

REJB reports receiving speaker honoraria from Eli Lilly and Springer Healthcare, sitting on the Novo Nordisk UK Foundation Research Selection Committee on a voluntary basis and acting as an independent advisor for Provent Bio, and has received speaking honoraria from Sanofi and Medscape that were donated to an education research fund. RS has received speaker honoraria for type 1 diabetes-related symposia sponsored by Sanofi. RMA is an employee and shareholder of Novo Nordisk A/S. PG’s institution has received grants from Novo Nordisk, Sanofi, Dexcom, Tandem, Medtronic, Abbott and Roche. In addition, PG has received consultancy fees from Abbott, Medtronic and Bayer; payments or honoraria for lectures, presentations, speakers’ bureaus, manuscript writing or educational events from Medtronic, Novo Nordisk, Abbott, Ypsomed, Vitalaire, Dexcom, Bayer and Insulet; support for attending meetings and/or travel from Sanofi, Novo Nordisk, Medtronic and Roche; and payments for participation on advisory boards from Insulet, Dexcom, Tandem and Ypsomed. Additionally, PG’s institution has received equipment including Dexcom CGMs for the ALERTT1 trial and Medtronic 780G devices for the CRISTAL trial from the respective sponsors. JV presented the EDENT1FI programme at the Medtronic industry symposium at the 61st Annual Meeting of the EASD in 2025. JV is a member of Sanofi’s Advisory Committee for the BR1DGE programme. JV also serves on the Steering Committee of Sanofi’s TH1NK Ahead initiative. The authors declare that there are no other relationships or activities that might bias, or be perceived to bias, their work.
