# Supplementary material for: Continuous glucose monitoring as a tool in early-stage type 1 diabetes
Source: Diabetologia. 2026 Mar 9;69(6):1413–31. doi: 10.1007/s00125-026-06707-4 (PMC13109206; doi:10.1007/s00125-026-06707-4)
Supplement: Supplementary file 1 — Slideset of figures (PPTX 326 KB) [file 125_2026_6707_MOESM1_ESM.pptx]

## Slide 1
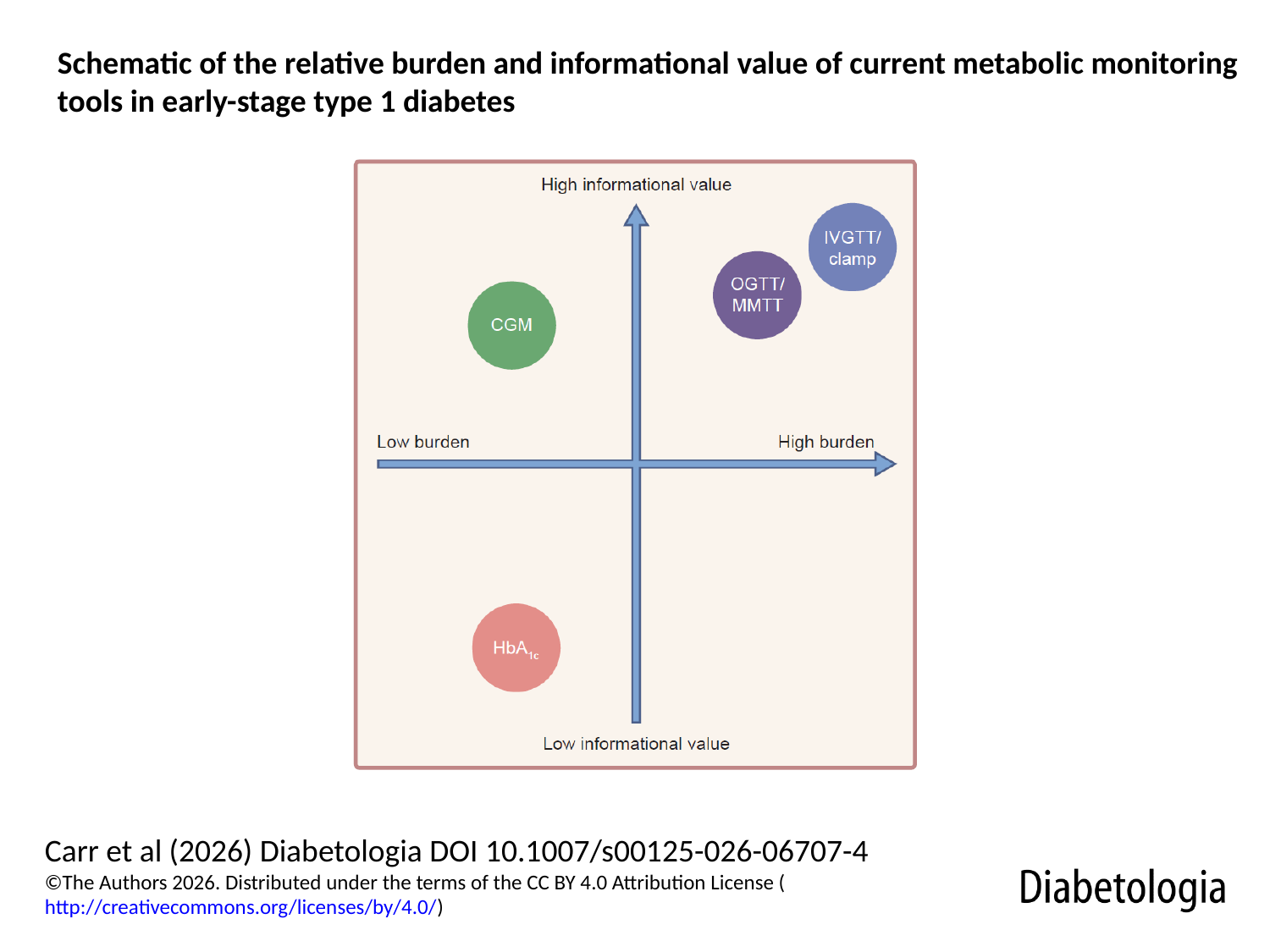

Schematic of the relative burden and informational value of current metabolic monitoring tools in early-stage type 1 diabetes
Carr et al (2026) Diabetologia DOI 10.1007/s00125-026-06707-4
©The Authors 2026. Distributed under the terms of the CC BY 4.0 Attribution License (http://creativecommons.org/licenses/by/4.0/)

## Slide 2
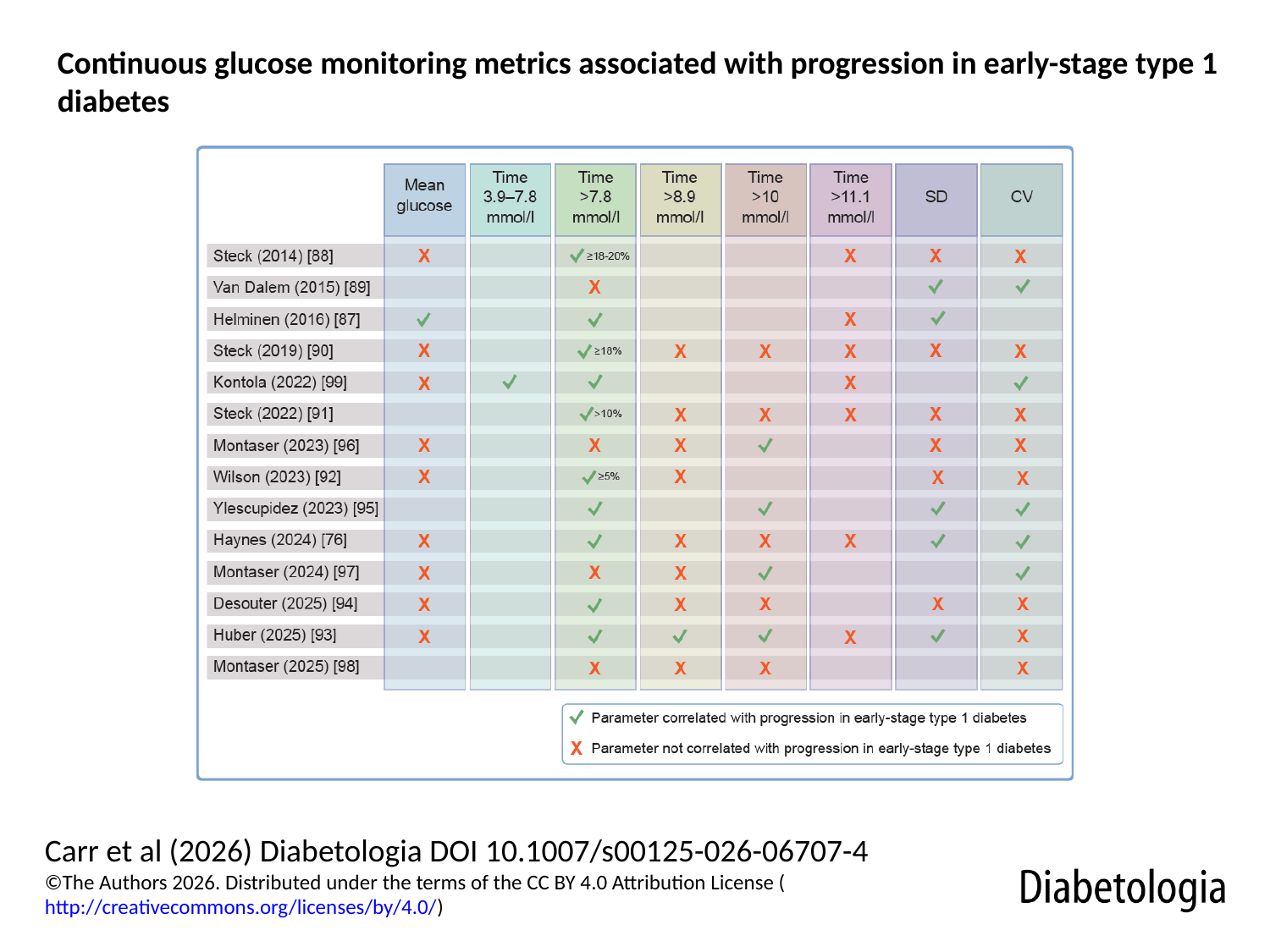

Continuous glucose monitoring metrics associated with progression in early-stage type 1 diabetes
Carr et al (2026) Diabetologia DOI 10.1007/s00125-026-06707-4
©The Authors 2026. Distributed under the terms of the CC BY 4.0 Attribution License (http://creativecommons.org/licenses/by/4.0/)

## Slide 3
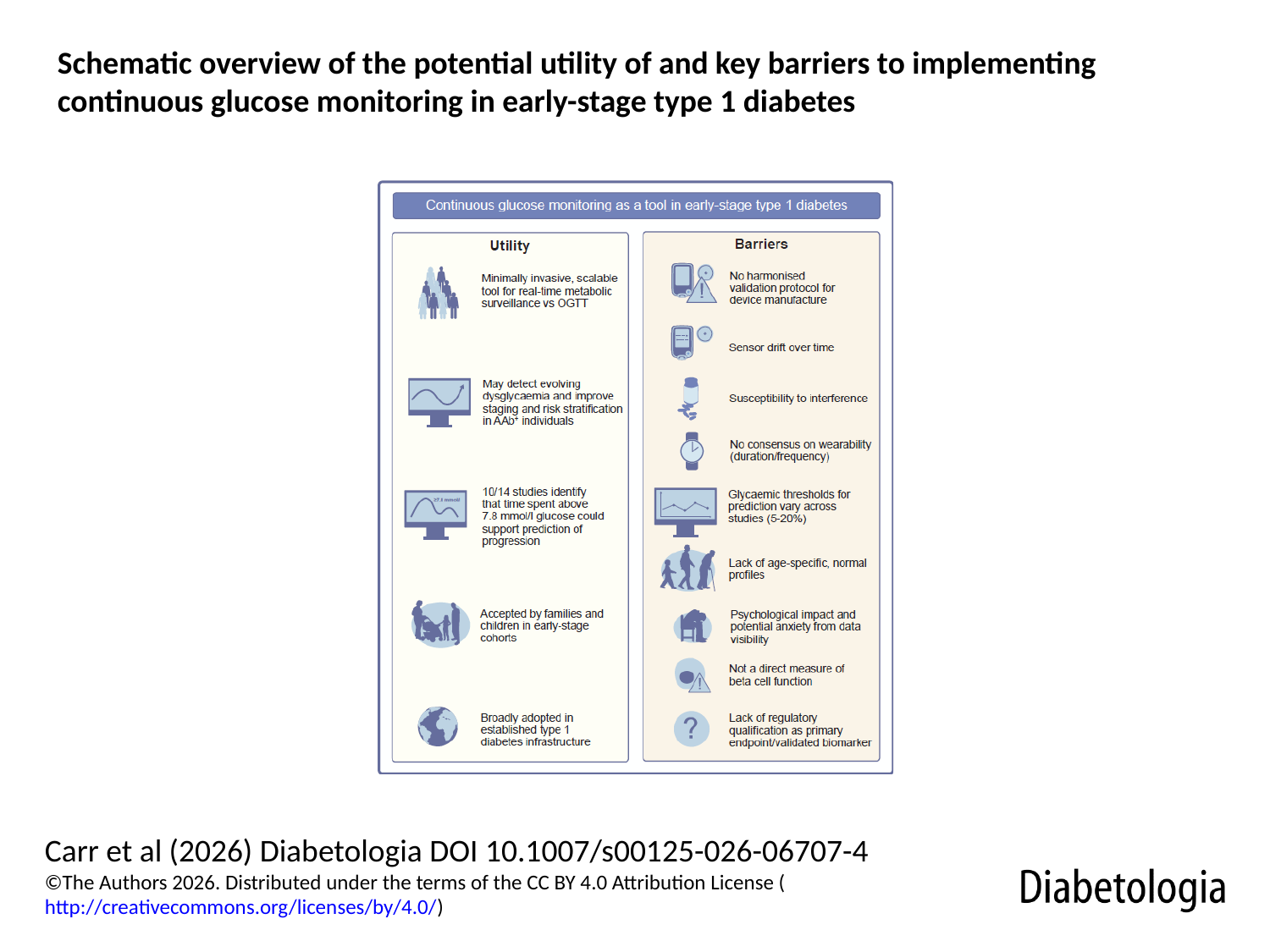

Schematic overview of the potential utility of and key barriers to implementing continuous glucose monitoring in early-stage type 1 diabetes
Carr et al (2026) Diabetologia DOI 10.1007/s00125-026-06707-4
©The Authors 2026. Distributed under the terms of the CC BY 4.0 Attribution License (http://creativecommons.org/licenses/by/4.0/)
